# Co-infectivity of hepatitis B virus and hepatitis E virus

**DOI:** 10.1186/1471-2334-12-S1-P1

**Published:** 2012-05-04

**Authors:** Akanksha Singh, Shiwani Singh, M Ahmad Ansari, M Irshad

**Affiliations:** 1 Dept. of Laboratory Medicine, All India Institute of Medical Sciences, Ansari Nagar, New Delhi-29, India

## Background

Viral hepatitis is a major health problem and is an important cause of morbidity and mortality throughout the world. Present study is aimed to assess the co-infection of hepatitis E virus with hepatitis B virus in HBV-DNA positive cases.

## Methods

A total number of 40 adult patients from various liver diseases with high level of ALT and AST were included in this study and were analyzed for hepatitis viral marker including HBsAg and IgM anti-HEV. The detection of HEV-RNA in 32 HBV-DNA positive cases was done by Real Time PCR.

## Results

The results of this study demonstrate that hepatitis E virus (HEV-RNA) infection to be rare (6.25%) in HBV-DNA positive cases. Surface antigen (HBsAg) for hepatitis B virus was positive in 18 cases (45%) and IgM Anti HEV was positive in 16 cases (40%). Hepatitis B virus infections, as the predominant causes of liver diseases, HBV-DNA was detected in 32 cases out of 40 cases and HEV-RNA was detected in 2 cases out of 32 HBV-DNA positive cases respectively. Co-infection of both HBV and HEV were reported in acute and chronic liver diseases in more than 18% cases by ELISA and 6.25% cases by real time PCR.

## Conclusion

HBV and HEV are the major cause of acute and chronic liver diseases. The data from this study indicates that presence of HEV-RNA in HBV-DNA positive cases, is rare in population. The co-infection of hepatitis B virus and hepatitis E virus in various liver diseases may occur.

